# Innovation and entrepreneurship among health professions students at a medical college in China: a cross-sectional study of attitudes, participation frequency, self-reported competencies, and associated factors

**DOI:** 10.3389/fmed.2026.1884275

**Published:** 2026-08-26

**Authors:** Liyuan Hou, Yuheng Zhang, Yifei Luan

**Affiliations:** 1 Savaid Stomatology School, Hangzhou Medical College, Hangzhou, China; 2 School of Clinical Medicine, Hangzhou Medical College, Hangzhou, Hangzhou, China; 3 School of Innovation and Entrepreneurship, Hangzhou Medical College, Hangzhou, China

**Keywords:** associated factors, health professions students, innovation and entrepreneurship attitude, participation frequency, self-reported innovation and entrepreneurship competency, training strategy

## Abstract

**Introduction:**

This cross-sectional study evaluated innovation and entrepreneurship (I&E) attitudes, participation frequency, and self-reported competencies among 706 undergraduate health professions students at Hangzhou Medical College and examined factors associated with self-reported competency scores.

**Methods:**

An open, voluntary online survey yielded 723 submissions. After 17 later duplicate submissions were removed by retaining each student’s first response, 706 unique participants were included. A study-specific 5-point questionnaire assessed I&E attitudes (7 items), participation frequency through a single-item behavioral indicator, and self-reported competencies (13 items). Split-sample exploratory and confirmatory factor analyses, internal-consistency analyses, group comparisons, and hierarchical linear regression were performed.

**Results:**

The mean scores were 3.95 ± 0.74 for I&E attitude, 3.37 ± 0.68 for self-reported competency, and 2.68 ± 0.90 for participation frequency. Exploratory factor analysis supported a two-factor attitude–self-reported-competency structure that explained 72.2% of the variance, and confirmatory factor analysis showed acceptable-to-good fit (CFI = 0.994, TLI = 0.993, RMSEA = 0.070, SRMR = 0.072). Students with prior I&E experience had higher self-reported competency scores than those without experience (3.47 ± 0.70 vs. 3.29 ± 0.65, *p* < 0.001), whereas the seven-item attitude scores did not differ significantly (*p* = 0.058). In the fully adjusted model, attitude (standardized *β* = 0.481, *p* < 0.001) and participation frequency (standardized *β* = 0.315, *p* < 0.001) were each associated with self-reported competency. The final model explained 41.8% of the variance, whereas the binary prior-experience variable was not associated with self-reported competency (standardized *β* = 0.005, *p* = 0.882).

**Conclusion:**

Students reported attitude scores above the scale midpoint, participation-frequency scores below the midpoint, and self-reported competency scores slightly above the midpoint. More favorable attitudes and more frequent participation were associated with higher self-reported competency scores; however, the cross-sectional design does not permit causal inference.

## 1 Introduction

In 2022, the Ministry of Education issued the “Opinions on Strengthening Organized Scientific Research in Universities to Promote High-level Self-reliance”, explicitly emphasizing the cultivation of innovative talents in interdisciplinary fields such as medicine-engineering and medicine-science integration. As the “Healthy China” strategy progresses, the healthcare industry is encountering unprecedented opportunities. Rapid advances in digital health, precision medicine, and technology-enabled care are reshaping health systems and increasing the need for graduates who can integrate clinical expertise with interdisciplinary, systems-oriented, and innovation-related competencies (1, 2). International medical-education standards further emphasize that curricula, assessment systems, educational resources, quality assurance, and governance should be responsive to local contexts and evolving health-system needs (3).

However, current medical training remains centered on clinical skills and basic scientific research, while I&E education often remains at a theoretical level. This discrepancy may result in a gap in which medical students possess the willingness to innovate but lack the necessary skills for project implementation, resource integration, and translation into practical solutions (4, 5). In China, “New Medical Science” is an educational reform initiative that promotes integration between medicine and disciplines such as engineering, information science, artificial intelligence, and the humanities. Internationally, medical schools have introduced I&E programs that integrate innovation, entrepreneurship, technology, leadership, health-systems science, the business of medicine, active learning, and interdisciplinary teaching (6). Against this background, students must not only master diagnostic techniques but also develop the ability to identify unmet clinical needs and translate them into feasible technological or organizational solutions—a role increasingly described as the “physician-innovator” or “physician-entrepreneur”. Previous studies have noted that, because of long academic cycles, heavy workloads, and relatively fixed career paths, the I&E awareness of medical students differs from that of students in other disciplines, and competency training often lacks targeted strategies (7, 8).

**Figure 1:**
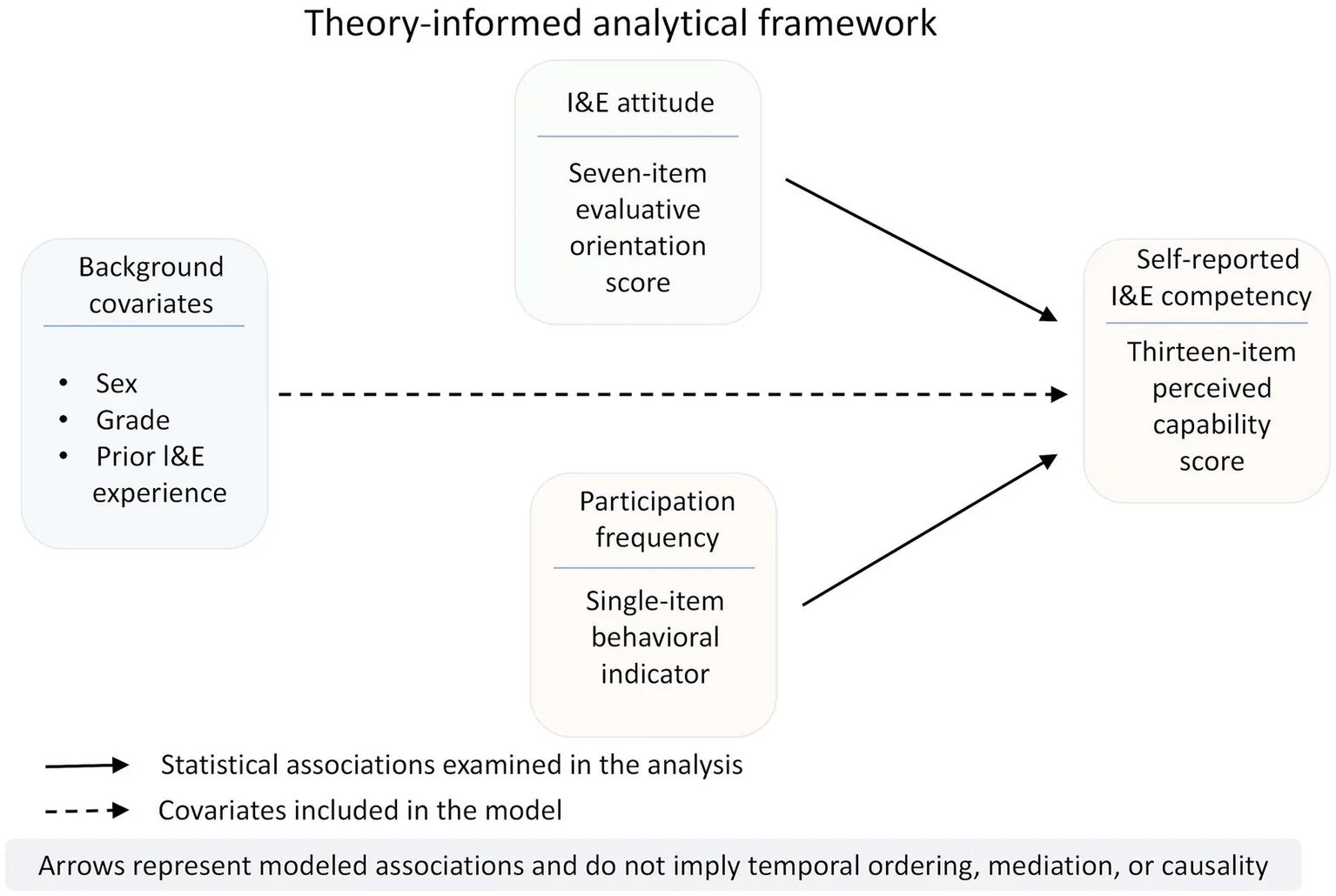
Theory-informed analytical framework for the cross-sectional study. I&E attitude was conceptualized as students’ evaluative orientation, participation frequency as the single behavioral indicator available in the questionnaire, and self-reported competency as perceived capability to perform I&E-related tasks. Sex, grade, and prior I&E experience were treated as background covariates. The framework informed construct specification and the organization of the hierarchical regression models. Solid arrows indicate associations evaluated in the fully adjusted model, whereas the dashed arrow indicates covariate adjustment. The arrows represent modeled associations and do not imply temporal ordering, mediation, or causality.

The measured constructs were interpreted within a theory-informed analytical framework drawing on the Theory of Planned Behavior and a constructivist learning perspective (9, 10). I&E attitude was conceptualized as students’ evaluative orientation toward innovation and entrepreneurship; participation frequency was treated as the single enacted behavioral indicator available in the questionnaire, and self-reported competency represented perceived capability to perform I&E-related tasks. The Theory of Planned Behavior informed the conceptual distinction between evaluative attitude and enacted participation, whereas a constructivist perspective provided a rationale for examining participation frequency in relation to perceived competency. Previous studies have already reported relationships among I&E attitudes, participation, and competency-related self-assessments (7, 8, 10). Accordingly, the contribution of the present study is primarily methodological: separating participation frequency from the attitude score, evaluating the questionnaire’s internal structure in independent exploratory and confirmatory subsamples, and estimating associations using effect-size reporting, multiplicity adjustment, sensitivity analyses, and HC3-robust hierarchical regression. Prior I&E experience was treated as a background exposure, while sex and grade were included as covariates. International evidence indicates that I&E education in the health professions commonly uses active, interdisciplinary, and experiential approaches, while digital education may broaden access but requires context-sensitive implementation and rigorous evaluation (11, 12). Because the questionnaire did not assess subjective norms, perceived behavioral control, or behavioral intention, the study was not designed as a complete test of the Theory of Planned Behavior. Figure 1 summarizes the analytical framework. Accordingly, we examined whether I&E attitude and participation frequency were associated with self-reported competency after adjustment for sex, grade, and prior I&E experience. Analyses involving prior experience and participant characteristics were considered exploratory.

## 2 Materials and methods

### 2.1 Study participants and recruitment

This single-institution cross-sectional survey was conducted from 14 to 31 March 2025. Undergraduate students at Hangzhou Medical College were invited to participate through an open invitation disseminated by student organizations. Eligible participants were full-time undergraduate students enrolled in medicine and allied health professions programs at Hangzhou Medical College. Participation was voluntary, no financial or academic incentive was offered, and participation was not linked to course grades, attendance, scholarships, awards, or other academic evaluation. A total of 723 submissions were received. Student identification numbers were used only to identify repeat submissions; 17 later duplicate submissions were removed, and the first response from each student was retained, yielding 706 unique participants. The final sample included 188 male students (26.63%) and 518 female students (73.37%). There were 5 fifth-year students (0.71%), 16 fourth-year students (2.27%), 81 third-year students (11.47%), 262 second-year students (37.11%), and 342 first-year students (48.44%). A total of 323 students (45.75%) reported prior I&E experience, whereas 383 (54.25%) reported no such experience. Participants were informed that identifying information would be used only for data verification, kept confidential, removed from the analytical dataset, and would not affect their academic standing. Electronic informed consent was obtained before participants accessed the questionnaire.

### 2.2 Ethical considerations

The study involving human participants was reviewed by the Medical Ethics Committee of Hangzhou Medical College. Because the study involved a minimal-risk educational survey, the committee determined that formal ethical approval was not required and waived the requirement for such approval. Electronic written informed consent was obtained directly from participants aged 18 years or older. For participants younger than 18 years, electronic written informed consent was obtained from a parent or legal guardian.

### 2.3 Research methods

#### 2.3.1 Questionnaire development and evaluation of internal structure and consistency

With reference to domains represented in relevant medical-education studies (13–15) and consideration of the institutional and health professions context, a study-specific questionnaire assessing health professions students’ I&E attitudes and self-reported competencies was developed. The questionnaire underwent three rounds of qualitative review by two medical-education experts, two I&E-education experts, and one senior clinical educator. The qualitative review was used to refine the conceptual domains, item wording, and feasibility of administration. A preliminary pilot involving several dozen students who were not included in the final sample was used to assess item comprehension and completion feasibility. Item-level expert relevance ratings and a detailed round-by-round revision record were not prospectively archived. Therefore, quantitative item-level and scale-level Content Validity Indices could not be validly reconstructed. Accordingly, the instrument is described as a study-specific questionnaire rather than a formally content-validated scale. The analyses reported below evaluate its internal structure and internal consistency in the present sample but do not constitute comprehensive validation of the instrument.

The questionnaire consisted of four parts: (1) basic information, including sex, age, grade, major, and prior I&E experience; (2) seven attitude items assessing willingness, interest, perceived need, perceived necessity, and perceived importance; (3) one participation-frequency item serving as a single behavioral indicator of how often students participated in I&E activities; and (4) 13 self-reported competency items covering opportunity identification, idea translation, risk response, resource integration, continuous learning, communication, business planning and execution, self-confidence, critical thinking and problem solving, policy knowledge, market research, data analysis, and leadership and organizational coordination. Prior I&E experience was recorded as a binary variable indicating participation in at least one I&E competition, university-level innovation or entrepreneurship project, or hospital-based innovation-translation activity. The questionnaire did not systematically record the specific activity type, number, duration, intensity, quality, or participant role. The complete question wording and response scales are provided in Supplementary Table S1.

### 2.4 Scoring criteria and construct specification

Each questionnaire item used a five-point ordered response scale tailored to its content. The willingness items ranged from 1 = very unwilling to 5 = very willing; interest ranged from 1 = very uninterested to 5 = very interested; perceived need ranged from 1 = not at all needed to 5 = very much needed; perceived necessity ranged from 1 = very unnecessary to 5 = very necessary; and perceived importance ranged from 1 = very unimportant to 5 = very important. Participation frequency was assessed using a single ordered-response item ranging from 1 = very infrequent to 5 = very frequent. This item represented the only direct indicator of enacted I&E participation available in the original questionnaire and was analyzed separately from the attitude score. Twelve self-reported competency items ranged from 1 = completely lacking to 5 = fully possessing, whereas knowledge of I&E support policies ranged from 1 = completely unfamiliar to 5 = fully familiar. Higher scores consistently indicated more favorable attitudes, more frequent participation, or stronger self-perceived competency or knowledge. The participation-frequency item was not intended to represent the broader multidimensional construct of student engagement, and an internal-consistency coefficient was not applicable to this single-item indicator.

### 2.5 Data collection and quality control

The questionnaire was distributed online via Wenjuanxing. All substantive items were mandatory, resulting in no missing item-level responses. Because the invitation was open and the number of students who viewed or received it was not recorded, a conventional response rate could not be calculated. The median completion time among the 706 unique participants was 82 s (interquartile range, 66–104 s). No *post hoc* response-time exclusion threshold was applied because such a threshold had not been prespecified; duplicate removal was the only record-level exclusion.

### 2.6 Statistical analysis

Analyses were conducted using Python 3.13 with the scipy, statsmodels, factor_analyzer, and semopy packages. The 706 unique participants were divided into exploratory and confirmatory subsamples (*n* = 353 each) using stratified random allocation by grade category and prior I&E experience. Exploratory factor analysis was based on a polychoric correlation matrix, minimum-residual extraction, and oblique rotation. Sampling adequacy was assessed using the Kaiser–Meyer–Olkin statistic and Bartlett’s test of sphericity; factor retention was guided by parallel analysis and interpretability. Confirmatory factor analysis was performed on the independent confirmatory subsample using the polychoric correlation matrix and unweighted least squares estimation. Model fit was evaluated using the comparative fit index (CFI), Tucker–Lewis index (TLI), root mean square error of approximation (RMSEA), and standardized root mean square residual (SRMR). Internal consistency was assessed using Cronbach’s *α* and McDonald’s *ω* for the seven-item attitude and 13-item self-reported competency domains. These coefficients were interpreted as evidence of internal consistency rather than evidence of content or structural validity. Evidence concerning the questionnaire’s internal structure was evaluated using EFA and CFA, while the latent-factor correlation and HTMT ratio were used to assess the empirical distinction between the attitude and self-reported competency constructs. Group comparisons used Welch’s t test or Welch’s ANOVA, with Games–Howell *post hoc* tests where appropriate. Item-level comparisons were adjusted using the Benjamini–Hochberg false-discovery-rate procedure. Hierarchical linear regression with heteroscedasticity-consistent HC3 standard errors examined factors associated with self-reported competency. Model 1 included sex, grade, and prior I&E experience; Model 2 added attitude, and Model 3 added participation frequency. The hierarchical ordering of variables reflected the theory-informed analytical framework: demographic and academic covariates together with prior experience were entered in Model 1, attitude was added in Model 2, and participation frequency was added in Model 3. This ordering was used to evaluate incremental changes in explained variance across the nested models. Results are reported as unstandardized coefficients, standardized *β* coefficients, 95% confidence intervals, and two-sided *p* values. A *p* value <0.05 was considered statistically significant. For grade-level group comparisons, fourth- and fifth-year students were combined because of their small cell sizes, whereas grade was retained as an ordinal variable coded 1–5 in the regression models. Sex and prior I&E experience were coded as binary variables (male/no = 0; female/yes = 1). Academic major was summarized descriptively. Major-specific inferential comparisons were not conducted because the individual program categories were unequal, several categories were relatively small, and such comparisons had not been prespecified. Model-comparison and sensitivity analyses were used to evaluate whether separating participation frequency from the attitude domain altered the questionnaire’s internal structure and the estimated association between attitude and self-reported competency. Sensitivity analyses comparing alternative attitude-score specifications are presented in Supplementary Table S5.

## 3 Results

### 3.1 Participant characteristics and outcome overview

The 706 unique participants had a mean age of 19.67 ± 1.22 years (range, 17–24 years); 518 (73.37%) were female, and 604 (85.55%) were in the first or second year. Participants were distributed across 21 identifiable medicine and allied health-professions programs; one additional eligible record contained an unclassifiable major response and was categorized as other/unclear in Table 1. Prior I&E experience was reported by 323 students (45.75%). The sample composition and the overall attitude, participation frequency, and self-reported competency scores are summarized in Table 1.

**Table 1:** Characteristics of the 706 unique study participants and overall outcome scores.

Characteristic	Category	*n* or summary	%
Sex	Male	188	26.63
Sex	Female	518	73.37
Grade	Year 1	342	48.44
Grade	Year 2	262	37.11
Grade	Year 3	81	11.47
Grade	Year 4	16	2.27
Grade	Year 5	5	0.71
Major	Clinical Medicine	92	13.03
Major	Rehabilitation Therapy	75	10.62
Major	Preventive Medicine	72	10.20
Major	Pharmacy	67	9.49
Major	Medical Imaging	63	8.92
Major	Nursing	61	8.64
Major	Stomatology	55	7.79
Major	Medical Laboratory Technology	45	6.37
Major	Medical Imaging Technology	39	5.52
Major	Health Inspection and Quarantine	31	4.39
Major	Anesthesiology	22	3.12
Major	Medical Information Engineering	15	2.12
Major	Food Quality and Safety	14	1.98
Major	Psychiatry	10	1.42
Major	Biotechnology	8	1.13
Major	Health Information Management	8	1.13
Major	Midwifery	7	0.99
Major	Intelligent Medical Engineering	7	0.99
Major	Biomedical Engineering	5	0.71
Major	Pediatrics	5	0.71
Major	Forensic Medicine	4	0.57
Major	Other/unclear	1	0.14
Prior I&E experience	Yes	323	45.75
Prior I&E experience	No	383	54.25
Age, years	Mean ± SD (range)	19.67 ± 1.22 (17–24)	–
I&E attitude (7 items)	Mean ± SD	3.95 ± 0.74	–
Participation frequency	Mean ± SD	2.68 ± 0.90	–
Self-reported I&E competency (13 items)	Mean ± SD	3.37 ± 0.68	–

I&E, innovation and entrepreneurship; SD, standard deviation. Academic-major labels were standardized from the program names recorded in the original questionnaire and summarized among the 706 unique participants. Fourth- and fifth-year students were grouped into a combined Years 4-5 category only for inferential grade-level comparisons.

### 3.2 Internal structure and internal consistency of the questionnaire

In the exploratory subsample, the Kaiser–Meyer–Olkin value was 0.935 and Bartlett’s test of sphericity was significant (*χ*^2^ = 8797.19, df = 190, *p* < 0.001). Parallel analysis supported two factors. The attitude and self-reported competency factors explained 27.4 and 44.7% of the variance, respectively, for a cumulative 72.2%. Primary factor loadings ranged from 0.698 to 0.971 for the seven attitude items and from 0.694 to 0.939 for the thirteen self-reported competency items.

In the independent confirmatory subsample, the final two-factor model showed better fit than both the original model that included participation frequency within the attitude domain and the one-factor model (*χ*^2^ = 461.65, df = 169, CFI = 0.994, TLI = 0.993, RMSEA = 0.070, SRMR = 0.072). By contrast, the one-factor alternative showed substantially poorer fit (CFI = 0.952, TLI = 0.947, RMSEA = 0.200, and SRMR = 0.144). After the retained model was refitted in the full analytical sample, the latent correlation between attitude and self-reported competency was 0.622, the HTMT ratio was 0.630, and standardized loadings ranged from 0.833 to 0.920 for attitude and from 0.793 to 0.898 for self-reported competency. Cronbach’s *α* was 0.944 for attitude and 0.958 for the self-reported competency domain, and McDonald’s *ω* was 0.964 and 0.968, respectively. Complete question wording and scoring are presented in Supplementary Table S1, exploratory and final confirmatory factor loadings in Supplementary Table S2, and confirmatory model comparisons in Supplementary Table S3. These findings provide sample-specific evidence concerning the proposed internal structure and internal consistency of the questionnaire; they do not establish content validity, criterion validity, test–retest reliability, responsiveness, cross-cultural validity, or external validity.

### 3.3 Overall I&E attitudes, participation frequency, and self-reported competencies

Among the 706 students, the mean attitude score was 3.95 ± 0.74, the mean participation frequency score was 2.68 ± 0.90, and the mean self-reported competency score was 3.37 ± 0.68. These continuous scores were retained for analysis rather than divided into overlapping or unvalidated categorical ranges. These overall outcome estimates are also presented alongside the participant characteristics in Table 1.

Regarding individual self-reported competency items, the three lowest-scoring items were “knowledge of I&E support policies” (3.04 ± 0.86), “ability to identify market opportunities” (3.13 ± 0.85), and “ability to translate ideas into products or services” (3.15 ± 0.85). The relatively higher-scoring items were “communication skills” (3.67 ± 0.81) and “learning ability to continuously update knowledge and skills” (3.62 ± 0.82).

### 3.4 Comparison of I&E attitudes, participation frequency, and self-reported competencies

Overall comparisons by sex and prior I&E experience are summarized in Table 2; grade-level comparisons are presented in Table 3; and item-level comparisons by prior I&E experience are presented in Tables 4 and 5.

**Table 2:** Comparisons of attitude, participation frequency, and self-reported competency by sex and prior I&E experience.

Grouping variable	Outcome	Reference group	Comparison group	Mean difference (95% CI)	Welch *t*	*p*	Hedges *g*
Sex	I&E attitude (7 items)	Male (*n*=188) 3.73 ± 0.84	Female (*n*=518) 4.03 ± 0.68	0.30 (0.17–0.43)	4.41	<0.001	0.41
Sex	Participation frequency	Male (*n*=188) 2.61 ± 0.96	Female (*n*=518) 2.70 ± 0.88	0.09 (−0.07–0.24)	1.09	0.277	0.10
Sex	Self-reported I&E competency (13 items)	Male (*n*=188) 3.32 ± 0.76	Female (*n*=518) 3.39 ± 0.65	0.07 (−0.05–0.20)	1.16	0.247	0.11
Prior I&E experience	I&E attitude (7 items)	No (*n*=383) 3.90 ± 0.73	Yes (*n*=323) 4.01 ± 0.75	0.11 (−0.003–0.216)	1.90	0.058	0.14
Prior I&E experience	Participation frequency	No (*n*=383) 2.42 ± 0.89	Yes (*n*=323) 2.98 ± 0.82	0.56 (0.43–0.68)	8.66	<0.001	0.65
Prior I&E experience	Self-reported I&E competency (13 items)	No (*n*=383) 3.29 ± 0.65	Yes (*n*=323) 3.47 ± 0.70	0.17 (0.07–0.28)	3.39	<0.001	0.26

Positive mean differences indicate higher scores in the comparison group. Two-sided Welch t tests were used. CI, confidence interval; I&E, innovation and entrepreneurship. Mean differences, confidence intervals, test statistics, P values, and effect sizes were calculated from unrounded data; displayed means may therefore not subtract exactly to the reported mean differences.

**Table 3:** Grade-level comparisons of attitude, participation frequency, and self-reported competency.

Grade category	*n*	I&E attitude	Participation frequency	Self-reported competency
Year 1	342	4.05 ± 0.71	2.66 ± 0.95	3.42 ± 0.73
Year 2	262	3.94 ± 0.75	2.69 ± 0.88	3.36 ± 0.66
Year 3	81	3.68 ± 0.68	2.64 ± 0.78	3.27 ± 0.51
Years 4–5	21	3.54 ± 0.89	2.90 ± 0.70	3.10 ± 0.60

Values are mean ± SD. Welch ANOVA results were as follows: I&E attitude, *F* (3, 83.54) = 7.75, *p* < 0.001, partial *η*² = 0.033; participation frequency, *F* (3, 88.05) = 0.85, *p* = 0.472, partial *η*² = 0.002; and self-reported competency, *F* (3, 87.40) = 2.96, *p* = 0.037, partial *η*² = 0.010. Years 4 and 5 were combined because of small cell sizes (*n* = 16 and *n* = 5, respectively). Games–Howell pairwise comparisons are presented in Supplementary Table S4. I&E, innovation and entrepreneurship; SD, standard deviation.

**Table 4:** Item-level self-reported I&E competency scores according to prior I&E experience.

Self-reported competency item	No experience (*n* = 383)	Prior experience (*n* = 323)	Difference	Raw *p*	FDR-adjusted *p*	Hedges *g*
Ability to identify market opportunities	3.01 ± 0.80	3.28 ± 0.90	0.27	<0.001	<0.001	0.32
Ability to translate ideas into products or services	3.06 ± 0.81	3.26 ± 0.90	0.20	0.002	0.006	0.24
Ability to take risks and respond to challenges	3.26 ± 0.86	3.38 ± 0.91	0.12	0.068	0.072	0.14
Ability to integrate resources and establish partnerships	3.28 ± 0.81	3.40 ± 0.89	0.12	0.058	0.069	0.14
Ability to continuously update knowledge and skills	3.54 ± 0.81	3.72 ± 0.83	0.18	0.004	0.009	0.22
Communication skills	3.59 ± 0.82	3.76 ± 0.80	0.17	0.005	0.009	0.21
Ability to develop and execute business plans	3.26 ± 0.79	3.42 ± 0.88	0.17	0.010	0.014	0.20
Self-confidence	3.53 ± 0.85	3.64 ± 0.82	0.11	0.072	0.072	0.14
Critical thinking and problem-solving skills	3.54 ± 0.78	3.67 ± 0.79	0.13	0.028	0.036	0.17
Knowledge of I&E support policies	2.96 ± 0.83	3.14 ± 0.88	0.18	0.005	0.009	0.22
Market research capability	3.12 ± 0.82	3.35 ± 0.86	0.23	<0.001	0.002	0.28
Data collection, organization, and analysis skills	3.42 ± 0.77	3.59 ± 0.83	0.17	0.006	0.010	0.21
Leadership and organizational coordination skills	3.28 ± 0.78	3.49 ± 0.86	0.20	0.001	0.005	0.25

Differences were calculated as the prior-experience group minus the no-experience group; positive values indicate higher scores among students with prior I&E experience. Benjamini–Hochberg false-discovery-rate adjustment was applied across the 13 comparisons. FDR, false discovery rate; I&E, innovation and entrepreneurship.

**Table 5:** Item-level I&E attitude and participation frequency scores according to prior I&E experience.

Domain	Item wording	No experience	Prior experience	Difference	Raw *p*	FDR *p*	Hedges *g*
Attitude	Willingness to participate in I&E activities	3.98 ± 0.84	4.07 ± 0.95	0.09	0.209	0.234	0.10
Attitude	Willingness to attend systematic I&E courses	3.85 ± 0.87	3.97 ± 0.91	0.12	0.072	0.144	0.14
Attitude	Willingness to prepare business plans and project presentations	3.58 ± 0.93	3.70 ± 0.98	0.12	0.096	0.153	0.13
Attitude	Interest in innovative ideas in daily life	3.91 ± 0.79	4.05 ± 0.78	0.14	0.015	0.061	0.18
Attitude	Perceived need to improve I&E literacy	3.97 ± 0.81	4.08 ± 0.84	0.11	0.066	0.144	0.14
Attitude	Perceived necessity of I&E activities	3.88 ± 0.83	3.96 ± 0.88	0.08	0.234	0.234	0.09
Attitude	Perceived importance of cultivating innovative thinking and competencies	4.14 ± 0.76	4.22 ± 0.75	0.08	0.166	0.222	0.10
Participation frequency	Frequency of participation in I&E activities	2.42 ± 0.89	2.98 ± 0.82	0.56	<0.001	<0.001	0.65

Differences were calculated as the prior-experience group minus the no-experience group; positive values indicate higher scores among students with prior I&E experience. FDR adjustment was applied across the eight comparisons. FDR, false discovery rate; I&E, innovation and entrepreneurship.

### 3.5 Comparison by sex

Female students reported higher attitude scores than male students (4.03 ± 0.68 vs. 3.73 ± 0.84; *p* < 0.001), whereas self-reported competency scores (*p* = 0.247) and participation frequency (*p* = 0.277) did not differ significantly by sex. Detailed results are shown in Table 2. The mean attitude difference was 0.30 points (95% CI, 0.17–0.43; Hedges g = 0.41), indicating a small-to-moderate between-sex difference. By contrast, the effect sizes for self-reported competency (*g* = 0.11) and participation frequency (*g* = 0.10) were small.

### 3.6 Comparison by I&E practical experience

Students reporting prior I&E experience had higher self-reported competency scores than those without experience (3.47 ± 0.70 vs. 3.29 ± 0.65; *p* < 0.001) and substantially higher participation frequency (2.98 ± 0.82 vs. 2.42 ± 0.89; *p* < 0.001). In contrast, the difference in the seven-item attitude score was small and did not reach statistical significance (4.01 ± 0.75 vs. 3.90 ± 0.73; *p* = 0.058). After Benjamini–Hochberg false-discovery-rate adjustment, prior experience remained associated with 10 of the 13 self-reported competency items, whereas the participation-frequency item was the only non-competency item that remained significant. Detailed results are shown in Tables 2, 4, and 5. The self-reported competency difference was modest (mean difference, 0.17; 95% CI, 0.07–0.28; Hedges *g* = 0.26), whereas the difference in participation frequency was larger (mean difference, 0.56; 95% CI, 0.43–0.68; *g* = 0.65). Among the self-reported competency items that remained significant after FDR adjustment, the largest mean differences were observed for opportunity identification (0.27 points), market research (0.23 points), idea translation (0.20 points), and leadership and organizational coordination (0.20 points). Risk-taking, resource integration, and self-confidence were not significant after FDR correction. None of the seven attitudinal items remained significant after correction, whereas the participation frequency item did. The complete self-reported competency-item comparisons are presented in Table 4, and the seven attitude items and the participation frequency item are presented in Table 5.

### 3.7 Grade-level comparisons

Because fourth- and fifth-year students were few, they were combined into a senior category for exploratory comparisons. Attitude scores differed across grade categories (Welch *F* = 7.75, *p* < 0.001; partial *η*^2^ = 0.033). Games–Howell *post hoc* testing showed that both first-year and second-year students reported higher attitude scores than third-year students (*p* < 0.001 and *p* = 0.023, respectively). Self-reported competency scores differed modestly overall (Welch *F* = 2.96, *p* = 0.037), but no pairwise comparison remained significant. Participation frequency did not differ across grades (*p* = 0.472). These findings do not support a progressive increase in attitude or self-reported competency with advancing grade. Across year 1, year 2, year 3, and years 4–5, the mean attitude scores were 4.05 ± 0.71, 3.94 ± 0.75, 3.68 ± 0.68, and 3.54 ± 0.89, respectively; the overall effect was small (partial *η*^2^ = 0.033). The corresponding self-reported competency means were 3.42 ± 0.73, 3.36 ± 0.66, 3.27 ± 0.51, and 3.10 ± 0.60 (partial *η*^2^ = 0.010), while participation frequency showed negligible between-grade variation (partial *η*^2^ = 0.002). Detailed grade-level results are presented in Table 3, and the Games–Howell *post hoc* comparisons are provided in Supplementary Table S4.

### 3.8 Hierarchical regression analysis of factors associated with self-reported I&E competency

In Model 1, which included sex, grade, and prior I&E experience, prior experience was positively associated with self-reported competency (standardized *β* = 0.149, *p* < 0.001), whereas grade was inversely associated (standardized *β* = −0.132, *p* < 0.001). Model 2 had an *R*^2^ of 0.336, compared with 0.035 for Model 1 (Δ*R*^2^ = 0.301). In Model 2, attitude was associated with competency (standardized *β* = 0.571, *p* < 0.001), while prior experience remained associated with competency with a smaller standardized coefficient (*β* = 0.095, *p* = 0.004).

Model 3 had an *R*^2^ of 0.418, representing an increase of 0.082 relative to Model 2. Attitude (*b* = 0.444, 95% CI, 0.359–0.529; standardized *β* = 0.481, *p* < 0.001) and participation frequency (*b* = 0.238, 95% CI, 0.178–0.298; standardized *β* = 0.315, *p* < 0.001) were independently associated with competency. The binary prior-experience variable was not associated with competency in Model 3 (standardized *β* = 0.005, *p* = 0.882), whereas participation frequency remained associated with competency. All variance-inflation factors were no greater than 1.21. Specific results are shown in Table 6. Neither sex (*p* = 0.110) nor grade (*p* = 0.363) was associated with self-reported competency in the fully adjusted model, and the final adjusted *R*^2^ was 0.414. These coefficients describe cross-sectional associations and should not be interpreted causally.

**Table 6:** Hierarchical linear regression of factors associated with self-reported I&E competency.

Model	Variable	*b*	HC3 SE	95% CI	*β*	*t*	*p*	VIF	*r*²	Adj. *r*²
Model 1	Female sex (vs. male)	0.055	0.062	−0.066–0.176	0.036	0.89	0.376	1.01	0.035	0.030
Model 1	Grade level (1–5)	−0.111	0.031	−0.171–0.051	−0.132	−3.63	<0.001	1.03	0.035	0.030
Model 1	Prior I&E experience (yes vs. no)	0.203	0.050	0.104–0.302	0.149	4.03	<0.001	1.04	0.035	0.030
Model 2	Female sex (vs. male)	−0.098	0.053	−0.202–0.005	-0.064	-1.86	0.063	1.04	0.336	0.333
Model 2	Grade level (1–5)	−0.015	0.024	−0.061–0.031	−0.018	−0.64	0.524	1.08	0.336	0.333
Model 2	Prior I&E experience (yes vs. no)	0.130	0.045	0.042–0.218	0.095	2.90	0.004	1.05	0.336	0.333
Model 2	I&E attitude score	0.528	0.044	0.441–0.614	0.571	11.94	<0.001	1.08	0.336	0.333
Model 3	Female sex (vs. male)	−0.082	0.051	−0.183–0.019	−0.053	−1.60	0.110	1.04	0.418	0.414
Model 3	Grade level (1–5)	−0.021	0.023	−0.065–0.024	−0.025	−0.91	0.363	1.08	0.418	0.414
Model 3	Prior I&E experience (yes vs. no)	0.007	0.046	−0.083–0.097	0.005	0.15	0.882	1.15	0.418	0.414
Model 3	I&E attitude score	0.444	0.043	0.359–0.529	0.481	10.23	<0.001	1.18	0.418	0.414
Model 3	Participation frequency	0.238	0.031	0.178–0.298	0.315	7.78	<0.001	1.21	0.418	0.414

Outcome: mean 13-item self-reported competency score. Model 1 included sex, grade, and prior I&E experience; Model 2 added attitude; Model 3 added participation frequency. The model-level changes in explained variance were Δ*r*² = 0.301 for Model 2 vs. Model 1 and Δ*r*² = 0.082 for Model 3 vs. Model 2. HC3 heteroscedasticity-consistent standard errors were used. CI, confidence interval; HC3 SE, heteroscedasticity-consistent standard error; I&E, innovation and entrepreneurship; VIF, variance inflation factor. Grade was entered as an ordinal variable coded from 1 to 5.

## 4 Discussion

### 4.1 Profiles of I&E attitude, participation frequency, and self-reported competency

The mean I&E attitude score was 3.95 ± 0.74, which was above the midpoint of the five-point scale, whereas the participation frequency score was lower at 2.68 ± 0.90. This pattern is relevant to broader discussions of the competencies future medical graduates may require in a rapidly transforming healthcare environment (16). University promotion of I&E competitions and hospital encouragement of clinical innovation are potentially relevant contextual features; however, these factors were not measured, and their relationships with the observed scores cannot be evaluated in this study. Although female students reported more favorable attitudes, the absence of corresponding sex differences in participation frequency or self-reported competency indicates that attitudinal variation should not be interpreted as evidence of differences in enacted participation or skill.

In this single-institution sample, the mean self-reported I&E competency score was 3.37 ± 0.68, with comparatively lower scores for “knowledge of I&E support policies”, “ability to identify market opportunities”, and “ability to translate ideas into products or services”. Reviews of innovation-oriented medical-school curricula show that many programs combine didactic teaching with hands-on modules and capstone projects (17), and recent initiatives have described student exposure to entrepreneurship and innovation activities (18). The comparatively lower scores observed in this study identify candidate domains for future needs assessment and curriculum evaluation. These scores represent self-perceptions rather than objectively demonstrated performance. Prior experience was associated most consistently with opportunity identification, market research, idea translation, and leadership and organizational coordination, although the between-group differences were generally small.

### 4.2 Methodological separation of attitude and participation frequency

The analyses clarify the relationships among attitude, participation frequency, prior experience, and self-reported competency. Related associations among I&E attitudes, educational participation or exposure, and competency-related self-assessments have been reported previously (7, 8, 10). Accordingly, the contribution of the present study lies primarily in the methodological refinement of these relationships rather than in identifying a new directional association. Specifically, participation frequency was separated from the attitude score, the questionnaire’s internal structure was evaluated in independent exploratory and confirmatory subsamples, and the associations were examined using effect-size reporting, false-discovery-rate adjustment, sensitivity analyses, and HC3-robust hierarchical regression. In directly comparable sensitivity models that included the attitude score and the binary prior-experience variable, separating participation frequency from the attitude score reduced the standardized attitude coefficient from 0.603 to 0.563 (Supplementary Table S5). In the fully adjusted hierarchical model, which retained prior experience and additionally included sex, grade, and participation frequency, attitude (standardized *β* = 0.481) and participation frequency (standardized *β* = 0.315) were each associated with self-reported competency. Because attitude, participation frequency, and self-reported competency were reported by the same respondents during the same survey sitting, the magnitude of these coefficients may be inflated by common-method variance and correlated response tendencies and should therefore be interpreted cautiously. This pattern is broadly consistent with the Theory of Planned Behavior and constructivist accounts in which evaluative attitudes and active participation are conceptually related to behavioral and competency-related outcomes (9, 10). Related research among pharmacy undergraduates has also examined students’ attitudes toward integrating I&E programs into professional education (19). The group comparisons further support the distinction among these constructs: prior experience showed a medium-to-large association with participation frequency, only a small association with self-reported competency, and no clear association with the seven-item attitude score. In the fully adjusted model, participation frequency remained associated with self-reported competency, whereas the binary prior-experience indicator was not.

### 4.3 Grade-level findings require cautious interpretation

Grade-level analyses did not show a progressive increase in I&E attitudes or self-reported competencies. First- and second-year students reported higher attitude scores than third-year students, whereas self-reported competency showed only a modest overall grade difference without significant pairwise contrasts. Previous program-evaluation work has reported associations between structured I&E educational exposure and student-reported outcomes (8). The present analysis compared grade cohorts observed at a single time point. Only 21 participants were in years 4–5, and all grade-related findings should therefore be considered exploratory. The small effect sizes and absence of significant pairwise self-reported competency differences do not support a strong stage-based interpretation.

### 4.4 Educational implications for prospective evaluation

#### 4.4.1 Potential curriculum, practice, and incubation priorities

The comparatively lower scores for knowledge of I&E support policies, opportunity identification, and idea translation identify candidate domains for prospective needs assessment and curriculum evaluation. Institutions could evaluate educational pathways combining coursework, supervised practice, and project-incubation opportunities. Potential components include interdisciplinary “Medicine + I&E” courses, project-based learning, and practical assessments involving clinical solution proposals or business plans developed with clinical and industry guidance (20). Affiliated-hospital placements, supervised exposure to medical-technology organizations, seed-funding mechanisms, and structured links with university science parks could also be evaluated (21). These approaches were not examined in the present study and require prospective evaluation.

#### 4.4.2 Potential role of a dual-mentor model

Given the technical, ethical, regulatory, and commercial complexity of healthcare innovation, a dual-mentor model could be prospectively evaluated as one possible form of student support (22). A clinical mentor could address clinical relevance, feasibility, patient safety, and data integrity, while an innovation or entrepreneurship mentor could contribute expertise in market analysis, financing, intellectual property, and risk management. Faculty-development activities, including exposure to industry placements and healthcare-innovation forums, could similarly be evaluated. These possibilities represent educational approaches for future investigation and require prospective evaluation.

#### 4.4.3 Institutional context and opportunities for voluntary participation

Institutions seeking to expand voluntary I&E participation could consider informational, curricular, and organizational support. Examples include introducing students to physician-innovator, clinical-academic, and entrepreneurial career pathways through campus resources and alumni engagement (23, 24), and recognizing appropriate I&E activities within elective or co-curricular systems. Research in other health-professions education settings has identified educator motivation, mentorship capacity, and clinical teaching environments as relevant implementation factors (25, 26). Any institutional strategy should preserve voluntariness and should not equate participation in competitions with objectively demonstrated competency.

## 5 Limitations and prospects

This study has several limitations. First, participants were recruited through an open voluntary invitation at a single medical college, limiting representativeness and introducing potential self-selection. The sample included students from multiple academic programs that may differ in curricular content, clinical exposure, research infrastructure, interdisciplinary training, and access to I&E opportunities. Because the program categories were unequal and several were relatively small, major-specific inferential comparisons were not conducted, and unmeasured discipline-level heterogeneity may have influenced the observed scores and associations. In addition, 604 participants (85.55%) were first- or second-year students, while only 21 were in years 4–5; the findings therefore predominantly reflect students in the earlier stages of undergraduate training. Second, although responses were treated confidentially and were unrelated to academic evaluation, identifying information was initially collected for duplicate verification, and the survey was disseminated through institution-linked student-organization channels using an online survey platform; social-desirability bias cannot be excluded. Third, the questionnaire was study-specific. Expert review was qualitative, item-level relevance ratings and a detailed round-by-round revision record were not prospectively archived, and quantitative Content Validity Indices could not be calculated. Although the EFA, CFA, latent-factor correlation, HTMT ratio, and internal-consistency estimates provided evidence concerning the questionnaire’s internal structure in this sample, they do not constitute comprehensive validation. Criterion validity, test–retest reliability, responsiveness, cross-cultural validity, and external replication were not evaluated. Fourth, I&E attitude, participation frequency, and self-reported competency were obtained from the same respondents during the same survey sitting. This same-source, same-sitting design is likely to inflate the magnitude of the Model 3 associations through common-method variance, consistency motifs, and correlated response tendencies. The standardized coefficients may therefore overstate the strength of the underlying relationships, and the self-reported competency scores should not be interpreted as objectively demonstrated performance. Fifth, the binary prior-experience variable did not capture the type, number, duration, intensity, quality, or participant role. Sixth, participation frequency was assessed using one self-reported item. This indicator should be interpreted as a narrow measure of how often students participated in I&E activities rather than as a comprehensive measure of student engagement. Its internal consistency could not be assessed, and its content coverage was limited. Seventh, the median questionnaire completion time was 82 s (interquartile range, 66–104 s). Because no minimum completion-time threshold had been prespecified, no time-based exclusions were applied, and rapid responding in some cases cannot be excluded. Finally, the cross-sectional, between-cohort design does not support inference about temporal ordering, mediation, causal effects, or within-student developmental change over training; residual confounding by academic achievement, family background, personality, and access to educational resources also remains possible.

In conclusion, students at this institution reported attitude scores above the scale midpoint, participation-frequency scores below the midpoint, and self-reported competency scores slightly above the midpoint. Attitude and participation frequency were each associated with self-reported competency in the fully adjusted model, whereas the binary prior-experience indicator was not. The principal contribution of the study is methodological, including the separation of participation frequency from the attitude score, split-sample evaluation of the questionnaire’s internal structure, and the use of robust and sensitivity analyses. The observed relationships provide a methodologically refined clarification of prior work. Multi-institutional longitudinal and intervention studies using validated multidimensional instruments, repeated measurements, and objective competency assessments are needed to evaluate I&E-related educational outcomes.

## Data Availability

The original contributions presented in the study are included in the article/Supplementary material, further inquiries can be directed to the corresponding author.
