# Supplementary material for: Innovation and entrepreneurship among health professions students at a medical college in China: a cross-sectional study of attitudes, participation frequency, self-reported competencies, and associated factors
Source: Front Med (Lausanne). 2026 Aug 26;13:1884275. doi: 10.3389/fmed.2026.1884275 (PMC13553448; doi:10.3389/fmed.2026.1884275)
Supplement: Supplementary file 1 [file Table_1.docx]

Supplementary Table S1. Study-specific questionnaire domains, question wording, and scoring

| **Domain** | **Question wording** | **Response scale** |
| --- | --- | --- |
| I&E attitude | Willingness to participate in innovation and entrepreneurship activities | 5-point ordered scale anchored from 1 = very unwilling to 5 = very willing |
| I&E attitude | Willingness to attend systematic innovation and entrepreneurship courses | 5-point ordered scale anchored from 1 = very unwilling to 5 = very willing |
| I&E attitude | Willingness to prepare business plans and project presentations | 5-point ordered scale anchored from 1 = very unwilling to 5 = very willing |
| I&E attitude | Interest in innovative ideas in daily life | 1 = very uninterested to 5 = very interested |
| I&E attitude | Perceived need to improve innovation and entrepreneurship literacy | 1 = not at all needed to 5 = very much needed |
| I&E attitude | Perceived necessity of innovation and entrepreneurship activities | 1 = very unnecessary to 5 = very necessary |
| I&E attitude | Perceived importance of cultivating innovative thinking and competencies | 1 = very unimportant to 5 = very important |
| Participation frequency | Frequency of participation in innovation and entrepreneurship activities | 5-point frequency scale anchored from 1 = very infrequent to 5 = very frequent |
| Self-reported I&E competency | Ability to identify market opportunities | 5-point self-assessment scale anchored from 1 = completely lacking to 5 = fully possessing |
| Self-reported I&E competency | Ability to translate ideas into products or services | 5-point self-assessment scale anchored from 1 = completely lacking to 5 = fully possessing |
| Self-reported I&E competency | Ability to take risks and respond to challenges | 5-point self-assessment scale anchored from 1 = completely lacking to 5 = fully possessing |
| Self-reported I&E competency | Ability to integrate resources and establish partnerships | 5-point self-assessment scale anchored from 1 = completely lacking to 5 = fully possessing |
| Self-reported I&E competency | Ability to continuously update knowledge and skills | 5-point self-assessment scale anchored from 1 = completely lacking to 5 = fully possessing |
| Self-reported I&E competency | Communication skills | 5-point self-assessment scale anchored from 1 = completely lacking to 5 = fully possessing |
| Self-reported I&E competency | Ability to develop and execute business plans | 5-point self-assessment scale anchored from 1 = completely lacking to 5 = fully possessing |
| Self-reported I&E competency | Self-confidence | 5-point self-assessment scale anchored from 1 = completely lacking to 5 = fully possessing |
| Self-reported I&E competency | Critical thinking and problem-solving skills | 5-point self-assessment scale anchored from 1 = completely lacking to 5 = fully possessing |
| Self-reported I&E competency | Knowledge of innovation and entrepreneurship support policies | 5-point familiarity scale anchored from 1 = completely unfamiliar to 5 = fully familiar |
| Self-reported I&E competency | Market research capability | 5-point self-assessment scale anchored from 1 = completely lacking to 5 = fully possessing |
| Self-reported I&E competency | Data collection, organization, and analysis skills | 5-point self-assessment scale anchored from 1 = completely lacking to 5 = fully possessing |
| Self-reported I&E competency | Leadership and organizational coordination skills | 5-point self-assessment scale anchored from 1 = completely lacking to 5 = fully possessing |

The I&E attitude score was calculated as the mean of the seven attitude questions. Participation frequency was analyzed as a separate single-item behavioral indicator. The self-reported I&E competency score was calculated as the mean of the thirteen competency questions. The questionnaire was administered in Chinese; the English wording and response anchors presented here are translations of the original items.

Supplementary Table S2. Exploratory and confirmatory factor loadings supporting the retained two-factor structure

| **Domain** | **Question wording** | **EFA loading: attitude** | **EFA loading: self-reported competency** | **Communality** | **CFA standardized loading** |
| --- | --- | --- | --- | --- | --- |
| I&E attitude | Willingness to participate in innovation and entrepreneurship activities | 0.883 | -0.013 | 0.780 | 0.833 |
| I&E attitude | Willingness to attend systematic innovation and entrepreneurship courses | 0.933 | 0.006 | 0.871 | 0.915 |
| I&E attitude | Willingness to prepare business plans and project presentations | 0.698 | 0.182 | 0.520 | 0.898 |
| I&E attitude | Interest in innovative ideas in daily life | 0.801 | 0.112 | 0.654 | 0.909 |
| I&E attitude | Perceived need to improve innovation and entrepreneurship literacy | 0.971 | -0.073 | 0.947 | 0.889 |
| I&E attitude | Perceived necessity of innovation and entrepreneurship activities | 0.903 | 0.035 | 0.817 | 0.920 |
| I&E attitude | Perceived importance of cultivating innovative thinking and competencies | 0.911 | -0.021 | 0.831 | 0.860 |
| Self-reported I&E competency | Ability to identify market opportunities | -0.003 | 0.791 | 0.626 | 0.793 |
| Self-reported I&E competency | Ability to translate ideas into products or services | -0.008 | 0.867 | 0.752 | 0.841 |
| Self-reported I&E competency | Ability to take risks and respond to challenges | 0.130 | 0.767 | 0.605 | 0.849 |
| Self-reported I&E competency | Ability to integrate resources and establish partnerships | 0.078 | 0.843 | 0.718 | 0.898 |
| Self-reported I&E competency | Ability to continuously update knowledge and skills | 0.177 | 0.694 | 0.513 | 0.850 |
| Self-reported I&E competency | Communication skills | 0.129 | 0.737 | 0.561 | 0.815 |
| Self-reported I&E competency | Ability to develop and execute business plans | 0.031 | 0.874 | 0.765 | 0.889 |
| Self-reported I&E competency | Self-confidence | 0.079 | 0.797 | 0.641 | 0.826 |
| Self-reported I&E competency | Critical thinking and problem-solving skills | 0.031 | 0.808 | 0.653 | 0.851 |
| Self-reported I&E competency | Knowledge of innovation and entrepreneurship support policies | -0.126 | 0.857 | 0.751 | 0.743 |
| Self-reported I&E competency | Market research capability | -0.139 | 0.939 | 0.901 | 0.833 |
| Self-reported I&E competency | Data collection, organization, and analysis skills | -0.026 | 0.863 | 0.746 | 0.850 |
| Self-reported I&E competency | Leadership and organizational coordination skills | -0.027 | 0.883 | 0.781 | 0.847 |

EFA was conducted in the exploratory subsample (n = 353) using a polychoric correlation matrix, minimum-residual extraction, and oblique rotation. Overall KMO = 0.935; Bartlett χ²(190) = 8797.19, P < 0.001. The attitude and self-reported competency factors explained 27.4% and 44.7% of the variance, respectively (cumulative variance explained, 72.2%). CFA loadings are reported for the final model refitted in the full analytical sample (n = 706) after evaluation in the independent confirmatory subsample.

Supplementary Table S3. Confirmatory factor-analysis model comparisons in the confirmatory subsample (n = 353)

| **Model specification** | **No. of questions** | **χ²** | **df** | **CFI** | **TLI** | **RMSEA** | **SRMR** |
| --- | --- | --- | --- | --- | --- | --- | --- |
| Original two-factor model with participation frequency included in the attitude domain | 21 | 819.95 | 188 | 0.988 | 0.987 | 0.098 | 0.085 |
| Final two-factor model with participation frequency analyzed separately | 20 | 461.65 | 169 | 0.994 | 0.993 | 0.070 | 0.072 |
| One-factor alternative model | 20 | 2574.67 | 170 | 0.952 | 0.947 | 0.200 | 0.144 |
| Two-factor sensitivity model excluding the business-plan willingness question | 19 | 436.18 | 151 | 0.994 | 0.993 | 0.073 | 0.075 |

Models were fitted to polychoric correlation matrices using unweighted least squares. The final two-factor model was retained because it showed better fit than the original and one-factor alternative models after the single participation-frequency item was excluded from the attitude domain and analyzed separately. In the full sample, the retained model showed χ²(169) = 723.94, CFI = 0.995, TLI = 0.994, RMSEA = 0.068, and SRMR = 0.065; the latent-factor correlation was 0.622 and the heterotrait-monotrait ratio was 0.630.

Supplementary Table S4. Games–Howell pairwise comparisons across grade categories

| **Outcome** | **Comparison** | **Mean difference** | **df** | **P** | **Hedges g** |
| --- | --- | --- | --- | --- | --- |
| Attitude | Year 1 vs. Year 2 | 0.110 | 543.07 | 0.265 | 0.151 |
| Attitude | Year 1 vs. Year 3 | 0.365 | 124.66 | <0.001 | 0.520 |
| Attitude | Year 1 vs. Years 4–5 | 0.504 | 21.59 | 0.079 | 0.700 |
| Attitude | Year 2 vs. Year 3 | 0.256 | 146.24 | 0.023 | 0.347 |
| Attitude | Year 2 vs. Years 4–5 | 0.394 | 22.37 | 0.225 | 0.515 |
| Attitude | Year 3 vs. Years 4–5 | 0.138 | 26.36 | 0.909 | 0.190 |
| Self-reported competency | Year 1 vs. Year 2 | 0.060 | 583.97 | 0.713 | 0.086 |
| Self-reported competency | Year 1 vs. Year 3 | 0.155 | 165.99 | 0.118 | 0.223 |
| Self-reported competency | Year 1 vs. Years 4–5 | 0.322 | 23.71 | 0.118 | 0.445 |
| Self-reported competency | Year 2 vs. Year 3 | 0.094 | 170.66 | 0.536 | 0.149 |
| Self-reported competency | Year 2 vs. Years 4–5 | 0.261 | 24.04 | 0.258 | 0.395 |
| Self-reported competency | Year 3 vs. Years 4–5 | 0.167 | 27.89 | 0.655 | 0.312 |
| Participation frequency | Year 1 vs. Year 2 | -0.033 | 580.21 | 0.972 | -0.036 |
| Participation frequency | Year 1 vs. Year 3 | 0.016 | 142.31 | 0.999 | 0.017 |
| Participation frequency | Year 1 vs. Years 4–5 | -0.247 | 24.78 | 0.435 | -0.262 |
| Participation frequency | Year 2 vs. Year 3 | 0.049 | 149.03 | 0.964 | 0.057 |
| Participation frequency | Year 2 vs. Years 4–5 | -0.214 | 25.40 | 0.560 | -0.245 |
| Participation frequency | Year 3 vs. Years 4–5 | -0.263 | 34.04 | 0.451 | -0.341 |

Mean differences were calculated as the score for the first-listed group minus the score for the second-listed group. Pairwise comparisons followed the corresponding Welch ANOVA. Fourth- and fifth-year students were combined because of small cell sizes (n = 16 and n = 5, respectively); these exploratory comparisons should therefore be interpreted cautiously.

Supplementary Table S5. Sensitivity analyses addressing potential overlap between attitude, participation frequency, and self-reported competency

| **Attitude-score specification** | **Standardized β for attitude** | **P** | **Standardized β for prior I&E experience** | **P** | **R²** |
| --- | --- | --- | --- | --- | --- |
| Eight-question attitude score including participation frequency | 0.603 | <0.001 | 0.057 | 0.080 | 0.375 |
| Seven-question attitude score with participation frequency analyzed separately | 0.563 | <0.001 | 0.087 | 0.007 | 0.332 |
| Six-item sensitivity score additionally excluding the business-plan willingness item | 0.545 | <0.001 | 0.089 | 0.006 | 0.312 |

Each model included the specified attitude score and the binary prior-experience variable. Separating participation frequency from the attitude score reduced the attitude coefficient from 0.603 to 0.563. Additional exclusion of the business-plan willingness question yielded a similar coefficient (0.545), supporting retention of that question within the attitude domain and separate analysis of participation frequency.

Abbreviations: CFA, confirmatory factor analysis; CFI, comparative fit index; EFA, exploratory factor analysis; HTMT, heterotrait-monotrait ratio; I&E, innovation and entrepreneurship; KMO, Kaiser–Meyer–Olkin; RMSEA, root mean square error of approximation; SRMR, standardized root mean square residual; TLI, Tucker–Lewis index.
